# Greater travel distance to specialized facilities is associated with higher survival for patients with soft-tissue sarcoma: US nationwide patterns

**DOI:** 10.1371/journal.pone.0252381

**Published:** 2021-06-04

**Authors:** Tomohiro Fujiwara, Koichi Ogura, John Healey

**Affiliations:** 1 Department of Surgery, Orthopaedic Service, Memorial Sloan Kettering Cancer Center, New York, New York, United States of America; 2 Department of Orthopaedic Surgery, Okayama University Graduate School of Medicine, Dentistry, and Pharmaceutical Sciences, Okayama, Japan; Chang Gung Memorial Hospital and Chang Gung University, Taoyuan, Taiwan, TAIWAN

## Abstract

**Purpose:**

The survival impact of geographic access to specialized care remains unknown in patients with soft-tissue sarcomas (STS). This study aimed to clarify the association between the patient travel distance and survival outcome and investigate the factors lying behind it.

**Methods:**

A total of 34 528 patients with STS registered in the National Cancer Data Base, diagnosed from 2004–2016, were investigated.

**Results:**

Tumor stage correlated with travel distance: patients with metastatic disease stayed closer to home. However, the type of facility showed greatest variation: 37.0%, 51.0%, 73.5%, and 75.9% of patients with ≤10 miles, 10.1–50 miles, 50.1–100 miles, and >100 miles, respectively (*P*<0.001), had a sarcoma care at academic/research centers. On a multivariable analysis, reduced mortality risk was associated with longer (versus short) travel distance (>100 miles: HR = 0.877; P = 0.001) and management at academic/research (versus non-academic/research) centers (HR = 0.857; *P*<0.001). The greatest divergence was seen in patients traveling very long distance (>100 miles) to an academic/research center, with a 26.9% survival benefit (HR = 0.731; *P*<0.001), compared with those traveling short distance (≤10 miles; 95.4% living in metropolitan area) to a non-academic/research center. There was no significant correlation between travel distance and survival in patients who had care at academic/research centers, whereas a survival benefit of management at academic/research centers was observed in every group of travel distance, regardless of tumor stage.

**Conclusions:**

This national study demonstrated that increased travel distance was associated with superior survival, attributable to a higher proportion of patients receiving sarcoma care at distant academic/research centers. These data support centralized care for STS. Overcoming referral and travel barriers may enable more patients to be treated at specialized centers and may further improve survival rates for patients with STS, even when it imposes an increased travel burden.

## Introduction

Soft-tissue sarcomas (STS) are a heterogeneous group of rare cancers (incidence of 4–5 cases per 100 000 individuals, < 1% of all malignant tumors [1,2]). This rarity implies most physicians or pathologists have little experience diagnosis or treating STS [3,4]. Centralized care for STS has been advocated for several decades [4–6], but data to support these recommendations are lacking in the United States. Prior European studies have demonstrated that sarcoma care at a specialized sarcoma center is associated with decreased risk of tumor relapse and better survival [7]. In those medical systems patients who reside far from sarcoma institutes are required to travel long distances to receive the most effective sarcoma care. However, the influence of travel distance on sarcoma stage at presentation or patient survival remains to be elucidated in the US system.

Patients with malignant disease must overcome social, economic, psychological, and family barriers to obtain the diagnosis and treatment [8]. Travel burden is an important issue affecting patient access to and use of health care [9–11]. Several studies demonstrated that travel burden can delay diagnosis and influence the management of many common cancers [8,11–17]. However, most of these studies were performed using state-level data or smaller geographic units, or used a variety of definitions regarding travel distance including travel time calculated using country centroid coordinates and Google (n.d.) maps [18]. Additionally, the relationship between travel burden and survival outcomes has not been established in patients with STS.

In the light of these observations, we aimed to evaluate the impact of geographic access on sarcoma stage at presentation and survival in patients with STS. We hypothesized that increased travel distance would be associated with a more advanced stage at presentation and poor survival, which would be attributed to socioeconomic status using a national cohort of patients with STS in the United States.

## Methods

We retrospectively reviewed patients with STS diagnosed from 2004–2016 registered in the National Cancer Data Base (NCDB). The data for this manuscript were analyzed anonymously after receipt of the data (accessed on March 30, 2020). An exemption from Memorial Sloan Kettering Cancer Center’s IRB was received for both approval and consent (IRB X20-033). The authors do not have the right to publicly share the NCDB dataset used in this study with anyone not on the participant user file (PUF) agreement (number 2016.2834), per NCDB policy. The dataset used in our study may be requested from NCDB via the provided email address (NCDB_PUF@facs.org) after completing the PUF application process. The NCDB represents approximately 70% of all cancers diagnosed in the United States, including 25 million cancer cases from >1500 hospitals [19,20].

**Fig 1:**
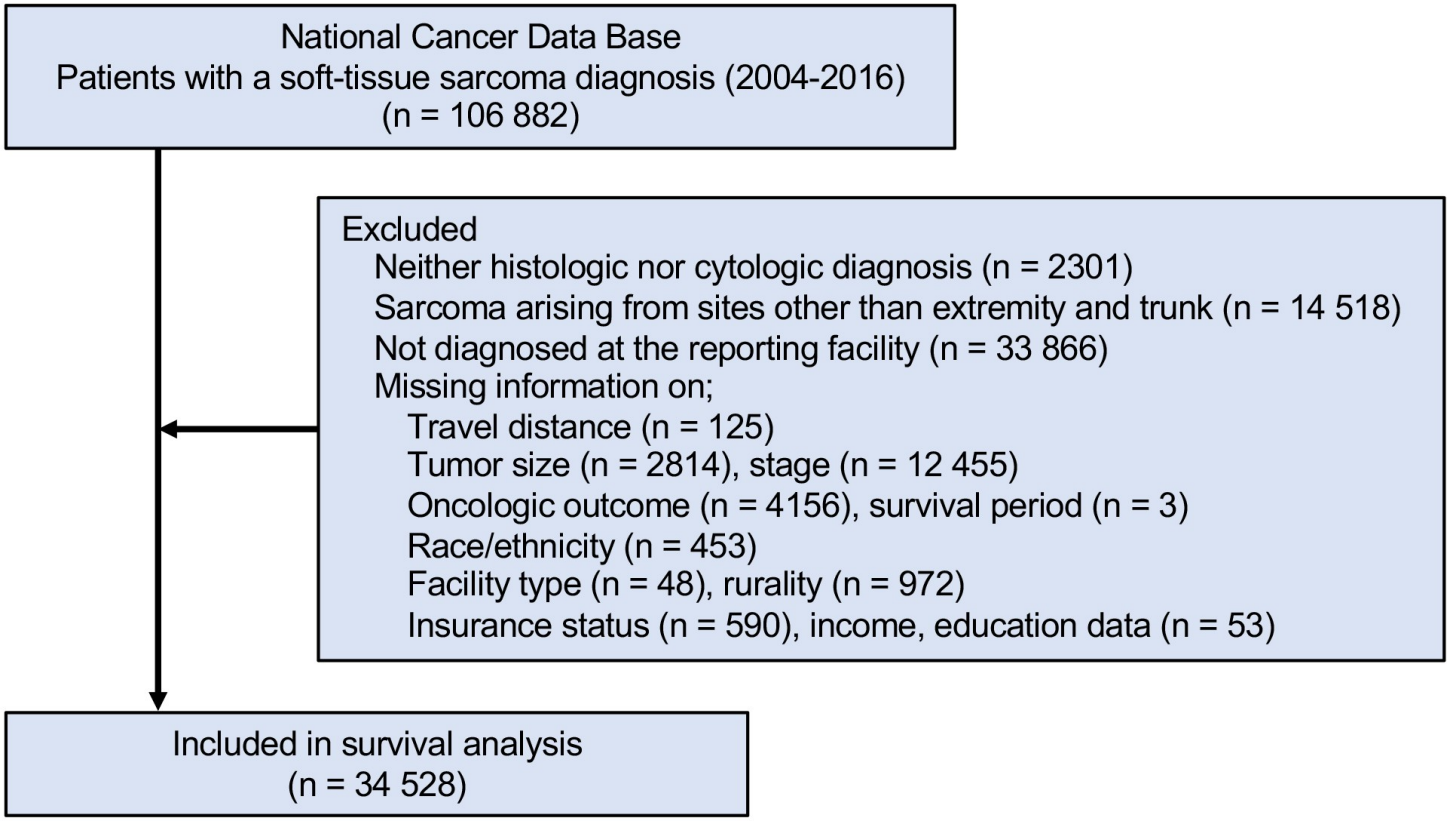
Flow diagram of study inclusion/exclusion criteria. NCDB, National Cancer Data Base.

The study flow diagram is shown in Fig 1. Patients who were not histologically diagnosed, defined as STS arising from sites other than extremity and trunk were excluded. Travel distance in the NCDB was calculated as the distance between the center of patient residential zip code and the address of the reporting hospital. Thus, patients diagnosed at institutions other than the reporting institute were excluded. Patients without a reported travel distance or any data required for analyses were also excluded.

The demographic and clinical data included age, sex, race, Charlson/Deyo comorbidity index [21], tumor size, tumor stage (American Joint Committee on Cancer [AJCC] staging system 7^th^ edition), indicators of income and education (defined as the estimated number of adults in a patient’s zip code who did not graduate from high school) based on area of residence derived from the 2012 American Community Survey data, insurance status, and rurality of residence. Hospital-level variables included institution type [22], and predefined geographic lesions. Patient travel distance was precalculated and provided in the NCDB, which represents the distance in miles between each patient’s residential zip code centroid and the reporting hospital’s zip code centroid. Travel distance were divided into quartiles on the basis of the literature [23–25]: short (≤10.0 miles); intermediate (10.1–50.0 miles); long (50.1–100.0 miles); very long (>100.0 miles). Differences in demographic, clinicopathological, socioeconomic, geographic, and hospital-level variables according to the travel distance were analyzed and survival impact of travel burden was estimated.

The study cohort comprised 34 528 patients with STS. Table 1 summarized patient demographics and clinical characteristics. Nearly half (47.5%) were diagnosed at an academic/research institute. The geographic distribution was South Atlantic (21.1%), followed by East North Central (17.8%), Middle Atlantic (15.1%), Pacific (12.9%), West North Central (8.6%), West South Central (7.8%), East South Central (6.4%), New England (5.2%), and Mountain region (4.9%). Nearly all patients received anti-cancer treatment at the institute where they were diagnosed (94.5%). The major histological diagnoses included undifferentiated pleomorphic sarcoma (UPS; n = 5097; 14.8%), leiomyosarcoma (n = 4588; 13.3%), atypical lipomatous tumor/well-differentiated liposarcoma (ALT/WDLS; n = 3800; 11.0%), myxofibrosarcoma (n = 1942; 5.6%), myxoid liposarcoma (n = 1727; 5.0%), and others (S1 Table).

**Table 1:** Patient demographic, clinical, and facility characteristics stratified by travel distance.

Characteristics	Total (n = 34 528)	≤10 miles (n = 16 895)	11–50 miles (n = 12 729)	51–100 miles (n = 2761)	≥101 miles (n = 2143)	*P* value
Age (median, years)	60 (IQR, 46–73)	62 (IQR, 47–75)	59 (IQR, 45–71)	59 (IQR, 44–71)	58 (IQR, 44–71)	<0.001
≤ 50	32.2%	30.1%	34.0%	34.2%	35.7%	
> 50	67.8%	69.9%	66.0%	65.8%	64.3%	
Sex						0.003
Male	52.8%	51.9%	53.2%	55.1%	54.2%	
Female	47.2%	48.1%	46.8%	44.9%	45.8%	
Race						<0.001
White	76.0%	70.2%	80.6%	83.7%	83.8%	
Black	12.8%	16.3%	10.0%	8.8%	7.2%	
Hispanic	7.4%	8.8%	6.3%	5.1%	5.6%	
Asian	2.6%	3.5%	2.1%	1.1%	1.3%	
Others	1.2%	1.2%	1.1%	1.3%	2.2%	
Comorbidity						<0.001
0	80.0%	79.0%	81.1%	79.2%	82.2%	
1	15.3%	15.6%	14.9%	16.1%	14.4%	
≥ 2	4.7%	5.3%	4.0%	4.7%	3.4%	
Size (cm)						<0.001
5.0	29.0%	31.3%	28.9%	21.0%	21.0%	
5.1–10.0	33.0%	32.6%	33.8%	33.2%	31.5%	
> 10.0	38.0%	36.1%	37.2%	45.8%	47.5%	
Stage (AJCC^a^ 7^th^ edition)						<0.001
I	37.8%	38.6%	37.5%	35.2%	35.5%	
II	21.5%	21.8%	21.7%	19.8%	19.5%	
III	27.1%	25.1%	27.6%	31.2%	34.4%	
IV	13.7%	14.5%	13.2%	13.7%	10.6%	
Diagnosis						<0.001
Undifferentiated pleomorphic sarcoma	14.8%	15.3%	14.5%	13.1%	13.9%	
Leiomyosarcoma	13.3%	14.6%	12.6%	11.1%	9.9%	
ALT/WLDS^b^	11.0%	10.5%	11.1%	11.7%	13.7%	
Myxofibrosarcoma	5.6%	5.2%	5.8%	7.2%	6.1%	
Myxoid liposarcoma	5.0%	4.7%	5.2%	5.4%	5.6%	
MPNST^c^	3.6%	3.3%	3.4%	5.1%	4.3%	
Dedifferentiated liposarcoma	3.3%	3.4%	3.2%	3.1%	2.8%	
Others	43.5%	43.0%	44.2%	43.2%	43.7%	
Center type						<0.001
Academic/research	47.5%	37.0%	51.0%	73.5%	75.9%	
Comprehensive community	32.1%	38.6%	29.9%	16.7%	14.0%	
Community	6.5%	8.7%	5.8%	1.1%	0.9%	
Other	13.9%	15.8%	13.3%	8.7%	9.2%	
Insurance						<0.001
Private	47.5%	44.6%	51.0%	47.8%	49.9%	
Medicare	38.7%	41.2%	36.2%	37.5%	36.0%	
Medicaid	8.1%	8.7%	7.1%	8.8%	8.0%	
Other government	1.1%	0.7%	1.3%	2.2%	2.3%	
Uninsured	4.6%	4.9%	4.5%	3.7%	3.7%	
Zip-code level income ($/year)						<0.001
< 38 000	16.9%	18.2%	11.2%	27.4%	26.6%	
38 000–47 999	22.2%	20.9%	19.6%	33.9%	32.9%	
48 000–62 999	26.8%	25.7%	28.5%	25.7%	26.4%	
≥ 63 000	34.1%	35.2%	40.6%	13.0%	14.0%	
Zip-code level education (%)^d^						<0.001
< 7.0	26.0%	27.6%	27.4%	15.0%	18.4%	
7.0 to 12.9	32.8%	31.9%	33.8%	32.5%	34.3%	
13.0 to 20.9	24.4%	22.6%	24.0%	32.7%	29.4%	
≥ 21.0	16.9%	17.9%	14.8%	19.9%	17.8%	
Rurality						<0.001
Metropolitan	86.4%	97.1%	84.7%	52.9%	55.9%	
Suburban	12.1%	2.9%	13.7%	41.3%	37.3%	
Rural	1.5%	0.0%	1.6%	5.9%	6.8%	
Center location						<0.001
Pacific	12.9%	14.9%	11.1%	9.5%	12.8%	
Mountain	4.9%	4.5%	4.7%	4.6%	9.3%	
West North Central	8.6%	7.2%	7.3%	14.5%	20.3%	
East North Central	17.8%	18.8%	18.5%	15.1%	9.9%	
Middle Atlantic	15.1%	17.8%	14.7%	9.6%	4.2%	
New England	5.2%	6.1%	5.2%	2.7%	1.8%	
West South Central	7.8%	6.6%	8.8%	8.3%	11.1%	
East South Central	6.4%	5.0%	6.9%	10.6%	9.3%	
South Atlantic	21.1%	19.1%	22.9%	25.1%	21.2%	
Treating/reporting status	94.5%	93.8%	94.9%	95.9%	96.1%	<0.001

^a^- AJCC = American Joint Committee on Cancer.

^b^-ALT/WDLS = atypical lipomatous tumor/well-differentiated liposarcoma.

^c^-MPNST = malignant peripheral nerve sheath tumor.

^d^-zip-code level education provides a measure of the number of adults in the patient’s zip code who did not graduate from high school.

Differences in covariables according to the travel distance were analyzed by using χ^2^ test. The Kaplan-Meier methods estimated overall survival (OS) and were compared by using log-rank test. Cox proportional hazards regression was used to account for the effects of patient, tumor, treatment, and hospital-level variables; all available covariates were included in the model. Two-sided *P* values were reported and were considered significant at *P* = 0.05. All statistical analyses were performed using SPSS software (version 23; IBM SPSS, Armonk, NY, USA).

## Results

Travel distance was distributed widely for each of the demographic and institutional factors (Table 1). In terms of an association between travel burden and stage at diagnosis, patients with advanced stage disease (stage IV) had a shorter travel burden for sarcoma care, compared with those with stage I–III disease: the proportion of patients with stage IV disease who traveled short (≤10 miles), intermediate (10.1 miles–50 miles), long (50.1–100 miles), and very long distance (>100 miles) was 51.6%, 35.6%, 8.0%, and 4.8%, respectively, while values for those with stage I–III disease were 48.5%, 37.1%, 8.0%, and 6.4%, respectively (*P* < 0.001).

Factors associated with OS were analyzed (Table 2). Adjusting for all measured factors, travel distance >100 miles (HR = 0.877; *P*<0.001) predicted lower risk of death compared with travel distance <10 miles. Other factors that were associated with improved OS in the multivariable analysis included younger age ≤50 years (>50 years: HR = 1.604 versus ≤50 years); female sex (HR = 0.934 versus male); Hispanic (HR = 0.937 versus white) and black race (HR = 0.856 versus white); low Charlson/Deyo comorbidity (score ≥2: HR = 1.653 versus score 1); small tumor size (10.1–15.0 cm: HR = 1.984 versus ≤5.0 cm); lower AJCC stage (stage IV: HR = 5.797 versus stage I); liposarcomas (ALT/WDLS: HR = 0.423; myxoid liposarcoma: HR = 0.558; dedifferentiated liposarcoma: HR = 0.757) and myxofibrosarcoma (HR = 0.627) versus UPS; diagnosed at an academic/research institute (HR = 0.857 versus non-academic/research); comprehensive community: HR = 1.186, community: HR = 1.208, other: HR = 1.110 versus academic/research); private insurance (Medicare: HR = 1.831 versus private); larger income ($48 000–62 999: HR = 1.075 versus ≥$63 000]; area where fewer people did not graduate from high school (13–20.9%: HR = 1.063 versus ≤7%); and the center location (Mountain: HR = 0.891 versus Pacific). Rural habitation was not statistically associated with OS (Table 2). In an analysis according to tumor stage, the 5-year overall survival was 76.6%, 62.9%, 43.1%, and 13.0% in patients with stage I, II, III, and IV disease, respectively (S1 Fig). In a multivariate analysis adjusting for all measured factors, very long travel distance (>100 miles) predicted a lower risk of death, compared with short travel distance (<10 miles), in both stage I–III (>100 miles: HR = 0.887; *P* = 0.008) and stage IV (>100 miles: HR = 0.826; *P* = 0.032) groups.

**Table 2:** Multivariate analysis with Cox regression hazard model adjusted for covariates to estimate the risk of overall mortality.

Covariate	Adjusted Hazard Ratio (95% CI)	*P* value
Age (years)		
≤50	Reference	
>50	1.604 (1.530–1.681)	<0.001
Sex		
Male	Reference	
Female	0.934 (0.903–0.965)	<0.001
Race		
White	Reference	
Hispanic	0.937 (0.887–0.989)	0.019s
Black	0.856 (0.793–0.924)	<0.001
Asian	0.922 (0.818–1.039)	0.184
Others	0.827 (0.700–0.977)	0.025
Comorbidity index		
0	Reference	
1	1.224 (1.173–1.277)	<0.001
≥ 2	1.653 (1.550–1.763)	<0.001
Tumor size (cm)		
≤ 5.0	Reference	
5.1–10.0	1.392 (1.323–1.463)	<0.001
10.1–15.0	1.984 (1.887–2.086)	<0.001
Stage (AJCC^a^ 7^th^ edition)		
I	Reference	
II	1.345 (1.274–1.419)	<0.001
III	1.878 (1.785–1.977)	<0.001
IV	5.797 (5.497–6.114)	<0.001
Diagnosis		
Undifferentiated pleomorphic sarcoma	Reference	
Leiomyosarcoma	0.999 (0.944–1.058)	0.981
ALT/WDLS	0.423 (0.388–0.461)	<0.001
Myxofibrosarcoma	0.627 (0.570–0.689)	<0.001
Myxoid liposarcoma	0.558 (0.501–0.622)	<0.001
MPNST	1.528 (1.397–1.670)	<0.001
Dedifferentiated liposarcoma	0.757 (0.689–0.832)	<0.001
Others	1.102 (1.053–1.154)	<0.001
Center type		
Academic/research	Reference	
Comprehensive community	1.186 (1.140–1.235)	<0.001
Community	1.208 (1.128–1.294)	<0.001
Other	1.110 (1.054–1.168)	<0.001
Insurance status		
Private	Reference	
Medicare	1.831 (1.760–1.905)	<0.001
Medicaid	1.359 (1.269–1.455)	<0.001
Other government	1.270 (1.072–1.504)	0.006
Uninsured	1.455 (1.337–1.584)	<0.001
Zip-code level income ($)		
≥ 63 000	Reference	
48 000–62 999	1.075 (1.004–1.150)	0.038
38 000–47 999	1.044 (0.986–1.104)	0.138
< 38 000	1.028 (0.979–1.079)	0.264
Zip code level education (%)		
<7%	Reference	
7%–12.9%	1.036 (0.987–1.088)	0.150
13%–20.9%	1.063 (1.003–1.127)	0.040
≥21%	1.052 (0.981–1.129)	0.156
Rurality		
Rural	Reference	
Suburban	1.019 (0.963–1.078)	0.514
Metropolitan	1.067 (0.936–1.216)	0.333
Facility location		
Pacific	Reference	
Mountain	0.891 (0.814–0.975)	0.012
West North Central	0.997 (0.926–1.073)	0.934
East North Central	0.978 (0.919–1.041)	0.479
Middle Atlantic	0.950 (0.890–1.014)	0.124
New England	0.943 (0.863–1.030)	0.191
West South Central	0.926 (0.857–1.000)	0.051
East South Central	1.022 (0.943–1.108)	0.596
South Atlantic	1.005 (0.946–1.068)	0.874
Travel distance (miles)		
≤10.0	Reference	
10.1–50.0	0.935 (0.900–0.971)	<0.001
50.1–100.0	0.959 (0.895–1.028)	0.240
>100.0	0.877 (0.810–0.949)	0.001

^a^- AJCC = American Joint Committee on Cancer.

**Fig 2:**
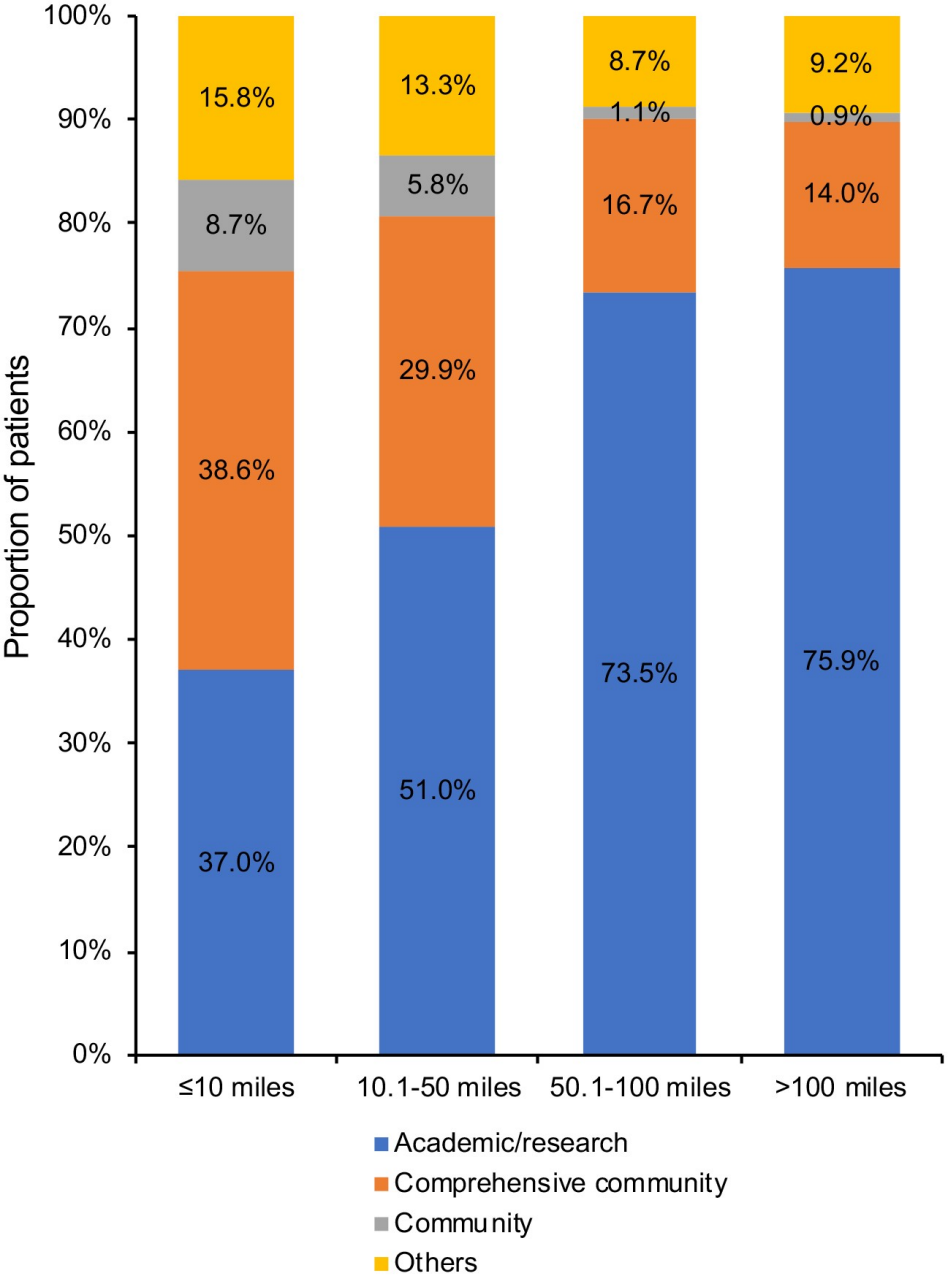
Proportion of patients who received a sarcoma care at academic/research center, comprehensive community cancer center, community cancer center, and other institutions, according to the travel distance.

The proportions of patients with favorable prognostic factors according to patient travel distance are shown in S2 Fig. Among these, the factor that showed greatest variation among four groups of travel distance was the type of facility (S2 Fig). Patients traveling longer distances were significantly more likely to be diagnosed and treated at an academic/research center (37.0%, 51.0%, 73.5%, and 75.9% for short, intermediate, long, and very long distance, respectively; *P*<0.001; Fig 2). In contrast, patients traveling shorter distances were significantly more likely to receive sarcoma care at a non-academic/research center (63.0%, 49.0%, 26.5%, and 24.1% for short, intermediate, long, and very long distance, respectively; *P*<0.001; Fig 2). In an analysis of the association between travel burden and type of facility according to tumor stage, patients who traveled longer distances were significantly more likely to be diagnosed and treated at an academic/research center in both the stage I–III (37.4%, 51.3%, 73.7%, and 75.8% for short, intermediate, long, and very long distance, respectively; *P* < 0.001; S3A Fig) and stage IV (34.6%, 48.8%, 71.5%, and 76.2%; *P* < 0.001; S3B Fig) groups.

**Fig 3:**
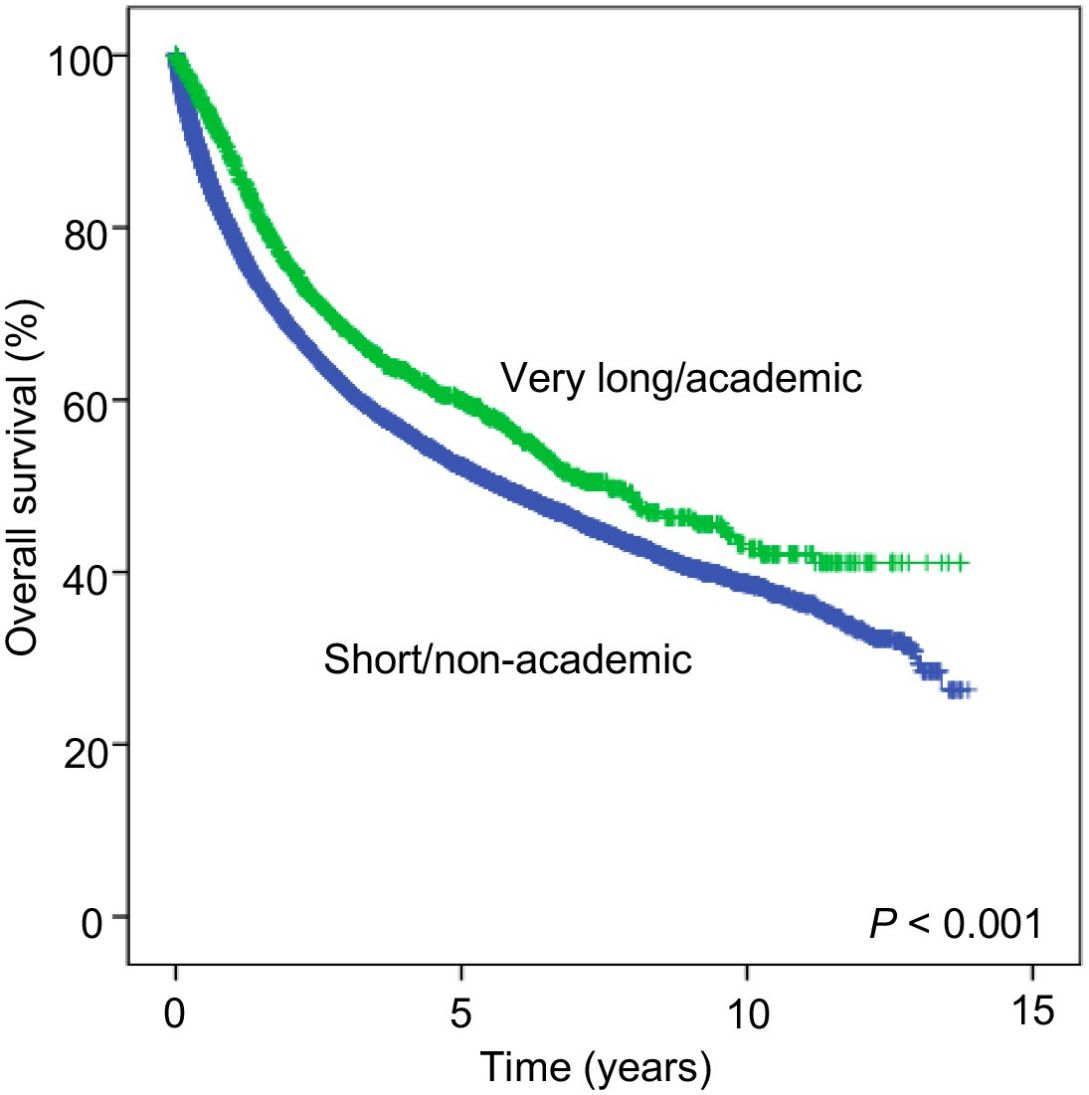
Kaplan-Meier curves showing overall survival for patients who traveled short distance (<10 miles) to an academic/research center versus those who traveled very long distance (>100 miles) to a non-academic/research center (*P* < 0.001; log-rank test).

The relationship between travel distance and institutional type was further analyzed. Patients who traveled short distances and received a diagnosis/treatment at a non-academic/research institution (short/non-academic) were compared to those who traveled very long distance and had a diagnosis/treatment at an academic/research institution (very long/academic). Table 3 summarizes patient demographic and clinical characteristics, stratified by the short/non-academic and very long/academic groups. Patients in the short/non-academic group were older (median, 64 years versus 58 years), less frequently White patients (76.7% versus 83.5%), more frequently had metastasis at diagnosis (stage IV; 14.7% versus 10.5%), and mostly lived in a metropolitan area (95.4% versus 56.3%) than those in the very long/academic group (Table 3). In an unadjusted comparison of survival between two groups, patients in the short/non-academic group had significantly worse survival outcomes than those in the very long/academic group (5-year OS; 52.3% vs 60.0%; *P*<0.001; Fig 3). Superior survival outcomes in the very long/academic group were also confirmed in patients who subsequently received anti-cancer treatment; the 5-year OS was 52.8% and 60.2% in the short/non-academic and very long/academic group, respectively (*P*<0.001; S4 Fig). After adjustment for relevant covariates, patients in the very long/academic group had a 26.9% survival benefit compared to those in the short/non-academic group (very long/academic: HR = 0.731) (Table 4). In an analysis according to tumor stage, patients in the very long/academic group had a 24.9% and a 34.0% survival benefit, compared with those in the short/non-academic group, with stage I–III (very long/academic: HR = 0.751 [95% CI, 0.673–0.837]; *P* < 0.001) and stage IV (very long/academic: HR = 0.660 [95% CI, 0.530–0.823]; *P* < 0.001) disease, respectively.

**Table 3:** Unadjusted characteristics of patients who traveled short distance to a non-academic/research center and patents traveling very long distance to an academic/research center.

	Short/non-academic (n = 10 642)	Very long/academic (n = 1 626)	*P* value
Age (median, years)	64 (IQR, 50–77)	58 (IQR, 42–69)	<0.001
≤ 50	26.4%	37.5%	
> 50	73.6%	62.5%	
Sex			0.078
Male	52.1%	54.1%	
Female	47.9%	45.9%	
Race			<0.001
White	76.7%	83.5%	
Black	12.3%	7.9%	
Hispanic	7.2%	5.4%	
Asian	2.6%	1.1%	
Others	1.1%	2.1%	
Comorbidity			<0.001
0	77.7%	83.7%	
1	16.5%	13.3%	
≥ 2	5.8%	3.0%	
Size (cm)			<0.001
≤ 5.0	33.5%	20.2%	
5.1–10.0	32.5%	32.0%	
> 10.0	34.0%	47.8%	
Stage (AJCC^a^ 7^th^ edition)			<0.001
I	38.5%	35.0%	
II	22.9%	20.1%	
III	24.0%	34.4%	
IV	14.7%	10.5%	
Diagnosis			<0.001
Undifferentiated pleomorphic sarcoma	15.9%	13.8%	
Leiomyosarcoma	15.7%	9.2%	
ALT/WDLS	9.9%	13.6%	
Myxofibrosarcoma	4.7%	5.8%	
Myxoid liposarcoma	4.2%	6.0%	
MPNST	2.8%	5.0%	
Dedifferentiated liposarcoma	3.5%	2.9%	
Others	43.3%	43.6%	
Insurance			<0.001
Private	43.7%	51.0%	
Medicare	45.4%	33.9%	
Medicaid	6.3%	8.4%	
Other government	0.6%	2.4%	
Uninsured	4.0%	4.2%	
Zip code level income ($/year)			<0.001
< 38 000	15.3%	25.9%	
38 000–47 999	21.9%	33.3%	
48 000–62 999	27.0%	26.4%	
≥ 63 000	35.9%	14.4%	
Zip-code level education			<0.001
< 7%	28.5%	19.3%	
7.0%–12.9%	34.8%	33.9%	
13.0%–20.9%	22.1%	29.6%	
≥ 21%	14.6%	17.1%	
Rurality			<0.001
Metropolitan	95.4%	56.3%	
Suburban	4.5%	37.1%	
Rural	0.1%	6.5%	
Center location			<0.001
Pacific	16.5%	13.2%	
Mountain	5.8%	7.9%	
West North Central	7.6%	22.4%	
East North Central	18.6%	9.8%	
Middle Atlantic	11.6%	4.7%	
New England	6.0%	1.2%	
West South Central	6.8%	7.3%	
East South Central	5.5%	10.8%	
South Atlantic	21.7%	22.8%	

^a^- AJCC = American Joint Committee on Cancer.

**Table 4:** Multivariate analysis with Cox regression hazard model adjusted for covariates to estimate the risk of overall mortality in patients with short travel/non-academic center and very long travel/academic center.

Covariate	Adjusted Hazard Ratio (95% CI)	*P* value
Age (years)		
≤50	Reference	
>50	1.714 (1.578–1.861)	<0.001
Sex		
Male	Reference	
Female	0.928 (0.880–0.979)	0.006
Race		
White	Reference	
Hispanic	0.883 (0.805–0.969)	0.009
Black	0.932 (0.823–1.055)	0.265
Asian	0.813 (0.658–1.005)	0.056
Others	0.820 (0.626–1.073)	0.148
Comorbidity index		
0	Reference	
1	1.242 (1.160–1.330)	<0.001
≥ 2	1.778 (1.612–1.962)	<0.001
Tumor size (cm)		
≤ 5.0	Reference	
5.1–10.0	1.364 (1.261–1.475)	<0.001
10.1–15.0	1.907 (1.763–2.063)	<0.001
Stage (AJCC^a^ 7^th^)		
I	Reference	
II	1.305 (1.199–1.420)	<0.001
III	1.928 (1.776–2.093)	<0.001
IV	5.861 (5.383–6.381)	<0.001
Diagnosis		
Undifferentiated pleomorphic sarcoma	Reference	
Leiomyosarcoma	0.984 (0.899–1.077)	0.724
ALT/WDLS	0.488 (0.426–0.559)	<0.001
Myxofibrosarcoma	0.587 (0.497–0.694)	<0.001
Myxoid liposarcoma	0.614 (0.513–0.735)	<0.001
MPNST	1.480 (1.265–1.733)	<0.001
Dedifferentiated liposarcoma	0.788 (0.677–0.917)	0.002
Others	1.155 (1.073–1.243)	<0.001
Insurance status		
Private	Reference	
Medicare	1.796 (1.684–1.915)	<0.001
Medicaid	1.426 (1.263–1.610)	<0.001
Other government	1.481 (1.084–2.022)	0.014
Uninsured	1.437 (1.240–1.666)	<0.001
Zip code level income ($/year)		
≥ 63 000	Reference	
48 000–62 999	1.076 (0.966–1.199)	0.185
38 000–47 999	1.064 (0.975–1.162)	0.164
< 38 000	1.038 (0.961–1.121)	0.341
Zip code level education (%)		
<7%	Reference	
7%–12.9%	1.069 (0.991–1.153)	0.084
13%–20.9%	1.076 (0.981–1.181)	0.119
≥21%	1.012 (0.903–1.134)	0.843
Rurality		
Rural	Reference	
Suburban	1.039 (0.936–1.153)	0.473
Metropolitan	1.136 (0.859–1.502)	0.372
Facility location		
Pacific	Reference	
Mountain	0.847 (0.741–0.968)	0.015
West North Central	1.025 (0.916–1.146)	0.667
East North Central	0.921 (0.837–1.012)	0.087
Middle Atlantic	0.973 (0.875–1.083)	0.620
New England	0.950 (0.830–1.087)	0.455
West South Central	0.960 (0.845–1.090)	0.525
East South Central	0.956 (0.840–1.089)	0.501
South Atlantic	1.049 (0.958–1.149)	0.298
Travel distance/center		
Short/non-academic	Reference	
Very long/academic	0.731 (0.663–0.806)	<0.001

^a^- AJCC = American Joint Committee on Cancer.

Further analysis of the association between travel burden and overall survival was performed, focusing on patients treated at academic/research centers. On multivariate analysis, there was no statistical difference in survival according to travel distance in patients who were treated at academic/research centers: 10–15 miles: HR = 0.967 (95% CI, 0.911 to 1.026), *P* = 0.268; 50–100 miles: HR = 1.002 (95% CI, 0.917 to 1.094), *P* = 0.969; >100 miles: HR = 0.906 (95% CI, 0.820 to 1.000), *P* = 0.051; versus ≤10.0 miles: HR = 1 (S2 Table). On the other hand, the multivariate analyses revealed that the survival benefit in patients treated at academic/research centers, compared with non-academic/research centers, was seen in every group of travel distance: patients with short, intermediate, long, and very long travel burden who received care at an academic/research institute had a survival benefit of 13.5%, 13.9%, 15.3%, and 20.1%, respectively, compared with those who had care at a non-academic institute (S3 Table).

In an analysis according to tumor stage, patients with stage I–III disease who received care at an academic/research institute had a 12.4% survival benefit, compared with those who had care at a non-academic institute (academic/research institute: HR = 0.874; *P* < 0.001). Similar results were observed in patients with stage IV disease: patients who received care at an academic/research institute had a 19.1% survival benefit, compared with those who had care at a non-academic institute (academic/research institute: HR = 0.809; *P* < 0.001).

## Discussion

Regionalization of diagnosis and treatment for STS at specialized centers has been advocated, but it would necessitate a greater travel burden for some patients [4,7]. In patients with carcinomas, travel burden has resulted in delays in diagnosis [8,11,13,17,26–30] and worse survival outcome [8,31–34]. To make this worthwhile, it is necessary to identify how the regionalized cases may differ from non-regionalized cases based on tumor stage or patient characteristics, how this translates into differences in patient survival, and if the effect is determined by care delivery by academic/specialized centers. However, the prognostic significance of travel burden is not established in STS patients. To our knowledge, this is the first demonstration of the relationship between geographic access and clinical outcomes in STS patients, showing that increased travel distance was associated with a better prognosis. Among several prognostic factors, a major difference according to travel distance was facility type; about 38% of patients with travel distance >10 miles received sarcoma care in a non-academic center, while >75% of patients with travel distance >100 miles were treated in an academic center. This result indicates facility type is a major factor associated with survival of patients with STS and supports regionalized specialized care.

The travel burden was related to tumor stage at diagnosis in patients with STS. Contrary to our hypothesis, short travel distance was associated with advanced stage at diagnosis. This relationship has varied based on diagnosis and region. In patients with breast cancer, three studies, in Kentucky, Illinois, and South Africa reported longer travel distance or poor geographical access was associated with advanced stage at diagnosis [27,28,35]. Yet, other studies in Virginia [12] and New Hampshire [36] described no association between travel burden and stage at diagnosis in breast cancer patients. In patients with colon cancer, results from two studies in Maine [29] and a large series of 296 474 patients registered in the NCDB [11], were consistent: patients who traveled longer distances were more likely to present with metastatic disease [11,29]. Our results were different from these studies: more patients with earlier stage STS, which could be regarded as operable, may be referred to specialized centers for sarcoma care, suggesting the centralized referral system does work for these patients. Further investigation about whether patients with other rare cancers, for which centralized care is advocated, are warranted.

Against our assumption, longer travel distances were associated with better patient survival. This result was different from previous investigations in other malignancies. For example, population-based study of 6848 patients with rectal cancer in Queensland, Australia, described that patients had a 6% increase in mortality risk for each 100-km increment in distance from the nearest radiotherapy center [31]. In a survival analysis of all cancer types, excluding sarcomas, in New South Wales, Australia, there was a 35% excess risk of dying from any cancer in the remote group compared to the highly accessible group [32]. The discrepancies in results between our study and these investigations indicate receiving the diagnosis and treatment in a specialized center is more important for survival than travel distance in patients with rare malignancies, such as STS.

Despite no difference in survival between rural and urban areas, patients living in small-urban and medium-urban areas had worse outcomes [33]. In a review of 476 patients with osteosarcoma registered in the Surveillance, Epidemiology and End Results Program Database, patients living in very rural areas had worse survival outcomes than those living in not very rural areas in Iowa, Utah, and New Mexico; there was no significant difference between rural and urban patients [18]. Our data, showing the results from the largest cohort of STS registered in the NCDB, were consistent with these reports in that urban/rural disparity does not correlate with patient survival. Additional studies analyzing the details of size of urban areas or rurality may produce information for STS patients in a specific area.

More than 75% of patients who traveled >100 miles received sarcoma care an academic/research center. Yet, about 38% of patients who traveled <10 miles received diagnoses/treatments at an academic/research center. Since diagnosis at an academic/research center is a favorable prognostic factor, the reason for better survival in patients with very long travel distance, as well as poorer survival in patients with short distance, could be explained by the differences of facility type where patients had sarcoma care. Our data indicate the importance of receiving diagnoses and treatments for STS at a specialized center, which strongly supports centralized care for STS. Our results were consistent with a previous investigation on retroperitoneal sarcomas; Schmitz et al. described five-year survival was better in patients with long travel to high-volume hospitals than patients with short travel to low-volume hospitals (63% versus 53%) [37]. In the present study, about 88% of patients who were diagnosed at a high-volume center with >12 cases per year received sarcoma care in an academic/research center. None of the comprehensive community or community cancer centers had cases >12 cases per year (*P* < 0.001; S4 Table, supporting the importance of receiving sarcoma care at a high-volume center. Our data showed patients with shorter distance more frequently travel to non-academic/research center. About 63% of patients with travel <10 miles, and about 50% of patients with intermediate travel distance from 10.1 miles to 50 miles, receive sarcoma care at a non-academic/research center. These patients are mostly (97.1% and 84.7% of patients with short and intermediate travel distance, respectively) living in a metropolitan area. The referring providers should know these data and reconsider their referral pattern and direct patients to a specialized center for sarcoma care, especially for those living in the metropolitan area.

Our study has several limitations. First, the details of the referral pathway in each patient are unavailable in the NCDB. We do not know whether the reporting center is the hospital where patients visited initially or as a referral institute. This information would contribute to further understanding of the prognostic significance of patient travel distance. Second, centers recognized as sarcoma centers are unavailable in the NCDB. Codes of the centers reporting each case are anonymized. This information, together with the details of the referral pathway, would clarify the current status of centralized care in the United States. Third, the NCDB does not collect full information on treatment, such as resection type (limb-salvage surgery or amputation), chemotherapy regimen, or dose of radiotherapy. However, the final status of surgical margins following the resection the primary tumor is provided. According to the registered information, negative surgical margin was achieved in 66.1% of surgically treated patients in short/non-academic group and 74.4% of patients in very long/academic group (*P* < 0.001; S5 Table). In patients with a positive margin, however, there was no statistical difference in the proportion of patients who received adjuvant radiotherapy between the two groups (short/non-academic, 37.1%; very long/academic, 34.0%; *P* = 0.324). Since surgical resection is the only known curative treatment for STS, and the status of resection margins is an important prognostic factor for survival [38–53], this information indicates more patients received appropriate care at an academic/research center. Fourth, 35% of cases were excluded because the diagnosis and reporting institutions differed. It is unknown how these cases corresponded to the larger population included in this report. They may or may not differ in prognostic, therapeutic, and outcome results, and should be studied separately. Travel distances was estimated using zip code centroids for both patients and diagnosing facilities, which raises possible selection bias. Finally, our results may not be fully generalizable, because the NCDB captures approximately 70% of incident cancers annually.

## Conclusions

The increased travel burden was associated with a superior survival outcome in patients with STS. This was attributed to higher proportion of receiving a sarcoma care at academic centers. Patients traveling longer distance to academic/research centers had a 26.9% survival benefit compared to those who traveled short distance to non-academic/research centers. Survival advantage was more significant when stratified by facility type, rather than travel distance, regardless of tumor stage, which confirmed the advantages of pursuing care at specialized centers. It is crucial referring providers to educate their patients about these data when giving recommendations about where to pursue care for STS.

## Supporting information

S1 Fig Kaplan-Meier curves showing overall survival stratified by tumor stage (*P* < 0.001; log-rank test). (PDF)

S2 Fig The proportion of patients with a favorable prognostic factor for overall survival according to the travel distance. (PDF)

S3 Fig Proportion of patients who received sarcoma care at an academic/research center, comprehensive community cancer center, community cancer center, and other institutions, according to travel distance. A. Patients with stage I–III disease. B. Patients with stage IV disease.
(PDF)

S4 Fig Kaplan-Meier curves showing overall survival for patients who subsequently received anti-cancer treatment in the short/non-academic and very long/academic groups (*P* < 0.001; log-rank test). (PDF)

S1 Table The histological diagnosis in the study cohort. (DOCX)

S2 Table Multivariate analysis with a Cox regression hazard model adjusted for covariates to estimate the risk of overall mortality in patients treated an academic/research center. (DOCX)

S3 Table Multivariate analysis with a Cox regression hazard model adjusted for covariates to estimate the risk of overall mortality according to travel distance. (DOCX)

S4 Table The type of facility and the number of patients registered to NCDB (*P* < 0.001; chi-square test). (DOCX)

S5 Table Surgical margins in the short/non-academic and very long/academic groups (*P* < 0.001; chi-square test). (DOCX)

## Abbreviations

AJCC: American Joint Committee on Cancer
MVA: multivariable
NCDB: National Cancer Database
OS: overall survival
STS: soft tissue sarcoma
