# Supplementary figures and images for: Greater travel distance to specialized facilities is associated with higher survival for patients with soft-tissue sarcoma: US nationwide patterns
Source: PLoS One. 2021 Jun 4;16(6):e0252381. doi: 10.1371/journal.pone.0252381 (PMC8177553; doi:10.1371/journal.pone.0252381)

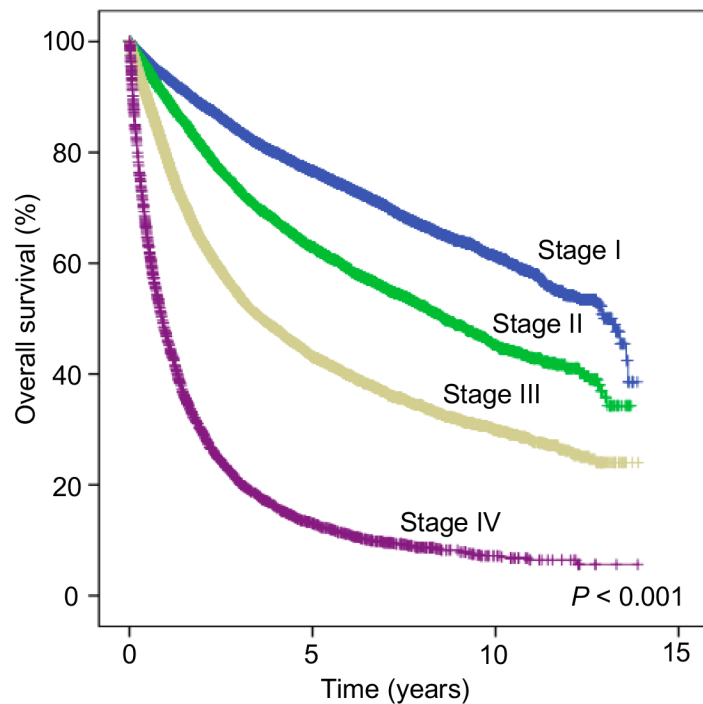

**S1 Fig.** Kaplan-Meier curves showing overall survival stratified by tumor stage ( $P < 0.001$ ; log-rank test).

Supplement: S1 Fig — (PDF) [file pone.0252381.s001.pdf]
