# Supplementary material for: Greater travel distance to specialized facilities is associated with higher survival for patients with soft-tissue sarcoma: US nationwide patterns
Source: PLoS One. 2021 Jun 4;16(6):e0252381. doi: 10.1371/journal.pone.0252381 (PMC8177553; doi:10.1371/journal.pone.0252381)
Supplement: S2 Fig — (PDF) [file pone.0252381.s002.pdf]

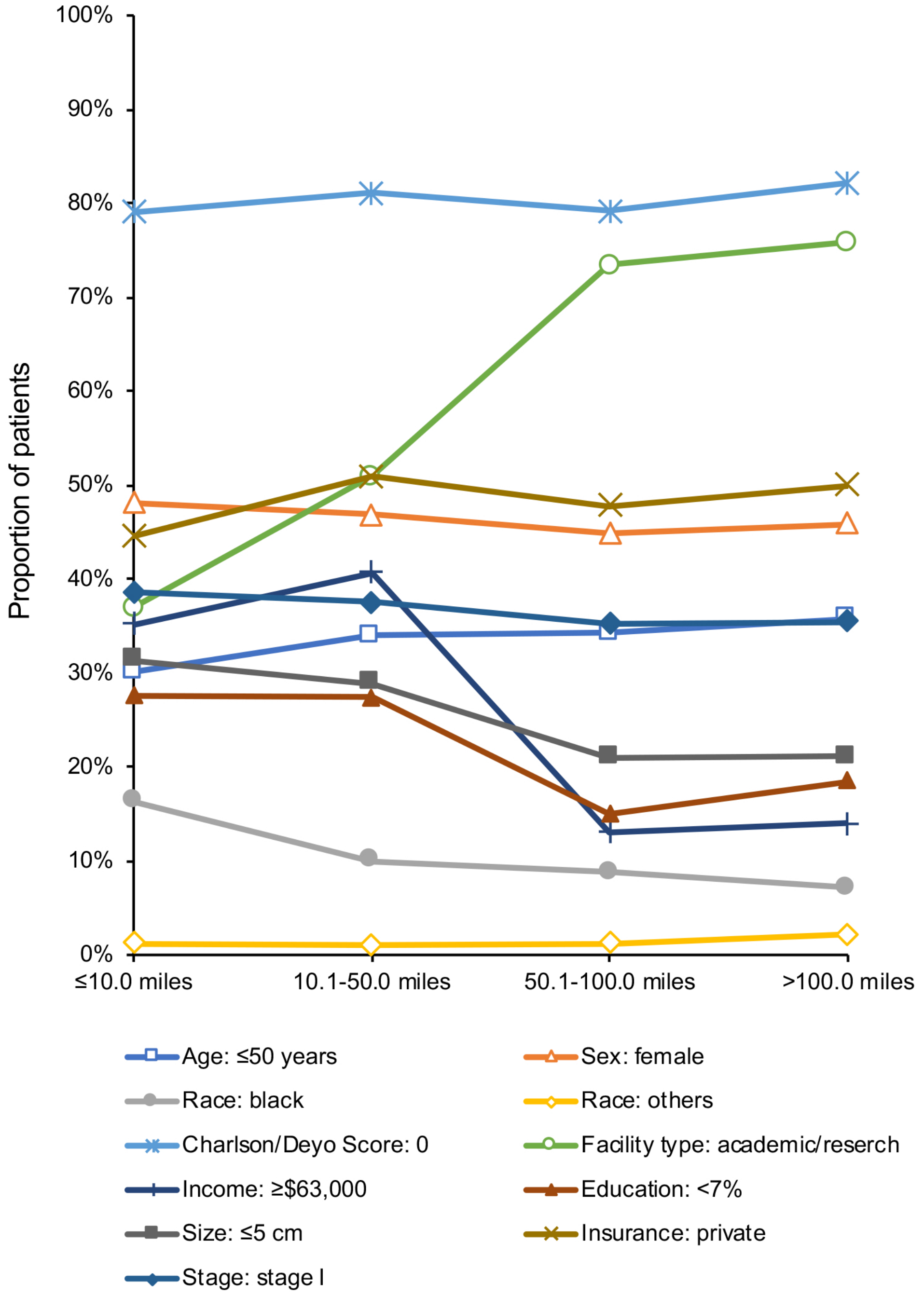

S2 Fig. The proportion of patients with a favorable prognostic factor for overall survival according to travel distance.
