# Supplementary material for: Greater travel distance to specialized facilities is associated with higher survival for patients with soft-tissue sarcoma: US nationwide patterns
Source: PLoS One. 2021 Jun 4;16(6):e0252381. doi: 10.1371/journal.pone.0252381 (PMC8177553; doi:10.1371/journal.pone.0252381)
Supplement: S3 Fig — A. Patients with stage I–III disease. B. Patients with stage IV disease. (PDF) [file pone.0252381.s003.pdf]

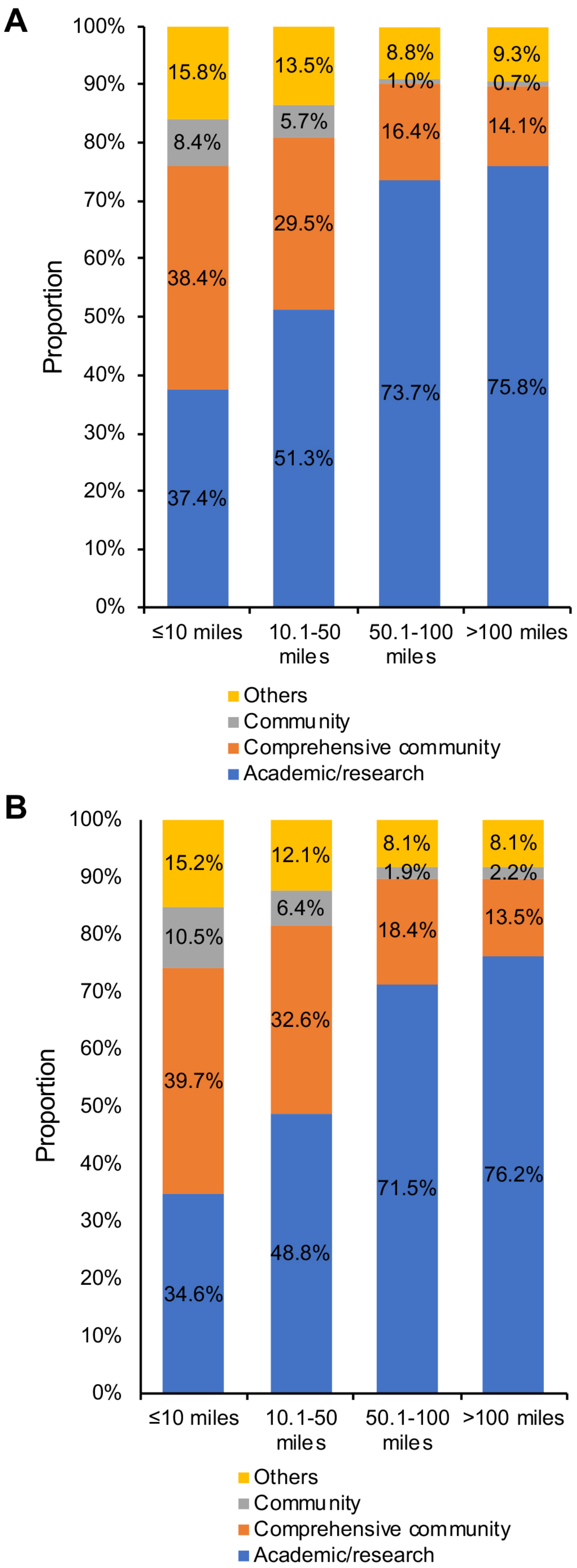

S3 Fig. Proportion of patients who received sarcoma care at an academic/research center, comprehensive community cancer center, community cancer center, or other institution, according to travel distance. A, Patients with stage I–III disease. B, Patients with stage IV disease.
