# Supplementary material for: Greater travel distance to specialized facilities is associated with higher survival for patients with soft-tissue sarcoma: US nationwide patterns
Source: PLoS One. 2021 Jun 4;16(6):e0252381. doi: 10.1371/journal.pone.0252381 (PMC8177553; doi:10.1371/journal.pone.0252381)
Supplement: S4 Fig — (PDF) [file pone.0252381.s004.pdf]

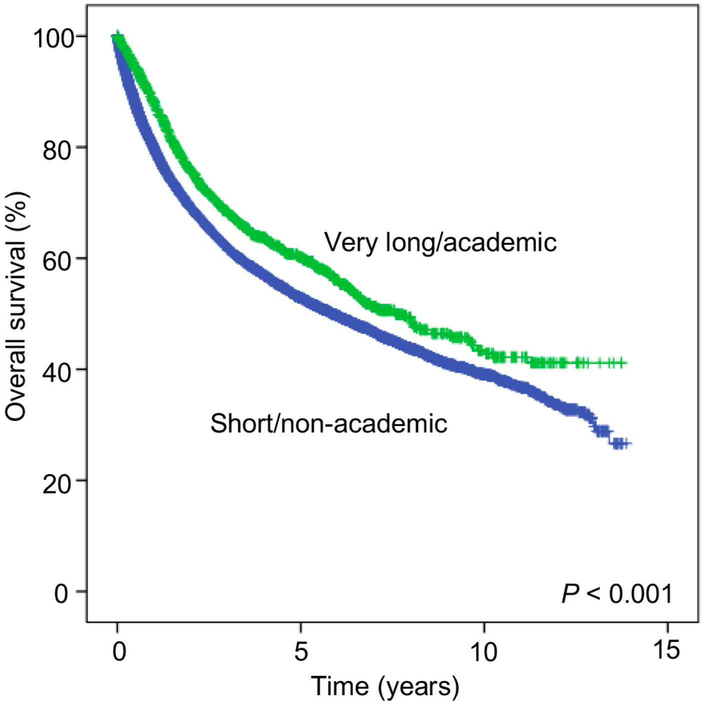

S4 Fig. Kaplan-Meier curves showing overall survival for patients who subsequently received anticancer treatment in the short/non-academic and very long/academic groups (*P*<0.001; log-rank test).
