# Supplementary material for: Greater travel distance to specialized facilities is associated with higher survival for patients with soft-tissue sarcoma: US nationwide patterns
Source: PLoS One. 2021 Jun 4;16(6):e0252381. doi: 10.1371/journal.pone.0252381 (PMC8177553; doi:10.1371/journal.pone.0252381)
Supplement: S1 Table — (DOCX) [file pone.0252381.s005.docx]

| **S1 Table**. The histological diagnosis in the study cohort | | |
| --- | --- | --- |
| Diagnosis | n | % |
| Undifferentiated pleomorphic sarcoma | 5,097 | 14.8% |
| Leiomyosarcoma | 4,588 | 13.3% |
| Atypical lipomatous tumor/well differentiated liposarcoma | 3,800 | 11.0% |
| Undifferentiated sarcoma | 3,480 | 10.1% |
| Myxofibrosarcoma | 1,942 | 5.6% |
| Myxoid liposarcoma | 1,727 | 5.0% |
| Undifferentiated spindle cell sarcoma | 1,505 | 4.4% |
| Synovial sarcoma | 1,458 | 4.2% |
| Malignant peripheral nerve sheath tumor | 1,226 | 3.6% |
| Dedifferentiated liposarcoma | 1,130 | 3.3% |
| Dermatofibrosarcoma protuberance | 1,012 | 2.9% |
| Angiosarcoma of soft tissue | 730 | 2.1% |
| Pleomorphic liposaroma | 610 | 1.8% |
| Adult fibrosarcoma | 580 | 1.7% |
| Epithelioid sarcoma | 417 | 1.2% |
| Exrtraskeletal Ewing sarcoma | 336 | 1.0% |
| Exrtraskeletal myxoid chondrosarcoma | 327 | 0.9% |
| Low-grade fibromyxoid sarcoma | 283 | 0.8% |
| Liposarcoma, not otherwise specified | 261 | 0.8% |
| Others | 4019 | 11.5% |
