# Supplementary material for: Greater travel distance to specialized facilities is associated with higher survival for patients with soft-tissue sarcoma: US nationwide patterns
Source: PLoS One. 2021 Jun 4;16(6):e0252381. doi: 10.1371/journal.pone.0252381 (PMC8177553; doi:10.1371/journal.pone.0252381)
Supplement: S2 Table — (DOCX) [file pone.0252381.s006.docx]

| **S2 Table**. Multivariate analysis with Cox regression hazard model adjusted for covariates to estimate the risk of overall mortality in patients treated an academic/research center | | |
| --- | --- | --- |
| Travel distance | Adjusted Hazard Ratio  (95% CI) | *P* value |
| ≤10.0 miles | Reference |  |
| 10.1–50.0 miles | 0.967 (0.911–1.026) | 0.268 |
| 50.1–100.0 miles | 1.002 (0.917–1.094) | 0.969 |
| >100.0 miles | 0.906 (0.820–1.000) | 0.051 |
