# Supplementary material for: Greater travel distance to specialized facilities is associated with higher survival for patients with soft-tissue sarcoma: US nationwide patterns
Source: PLoS One. 2021 Jun 4;16(6):e0252381. doi: 10.1371/journal.pone.0252381 (PMC8177553; doi:10.1371/journal.pone.0252381)
Supplement: S3 Table — (DOCX) [file pone.0252381.s007.docx]

| **S3 Table**. Multivariate analysis with Cox regression hazard model adjusted for covariates to estimate the risk of overall mortality according to the travel distance | | |
| --- | --- | --- |
| Travel distance/facility type | Adjusted Hazard Ratio  (95% CI) | *P* value |
| Short distance (≤10.0 miles) |  |  |
| Non-academic/research centers | Reference |  |
| Academic/research center | 0.865 (0.821–0.911) | <0.001 |
| Intermediate distance (10.1–50.0 miles) |  |  |
| Non-academic/research centers | Reference |  |
| Academic/research center | 0.861 (0.812–0.912) | <0.001 |
| Long distance (50.1–100.0 miles) |  |  |
| Non-academic/research centers | Reference |  |
| Academic/research center | 0.847 (0.739–0.972) | 0.018 |
| Very long distance (>100.0 miles) |  |  |
| Non-academic/research centers | Reference |  |
| Academic/research center | 0.799 (0.678–0.941) | 0.007 |
