# Supplementary material for: Greater travel distance to specialized facilities is associated with higher survival for patients with soft-tissue sarcoma: US nationwide patterns
Source: PLoS One. 2021 Jun 4;16(6):e0252381. doi: 10.1371/journal.pone.0252381 (PMC8177553; doi:10.1371/journal.pone.0252381)
Supplement: S4 Table — (DOCX) [file pone.0252381.s008.docx]

| **S4 Table**. The type of facility and the number of patients registered to NCDB (P < 0.001; chi-square test) | | | | | | |
| --- | --- | --- | --- | --- | --- | --- |
|  | ≥12 cases‎/year | | 4–11 cases‎/year | | ≤3 cases‎/year | |
|  | n | % | n | % | n | % |
| Academic‎/research | 6,765 | 87.8% | 7,165 | 59.7% | 2,463 | 16.6% |
| Comprehensive community | 0 | 0.0% | 3,270 | 27.3% | 7,812 | 52.7% |
| Community | 0 | 0.0% | 71 | 0.6% | 2,184 | 14.7% |
| Others | 944 | 12.2% | 1,485 | 12.4% | 2,369 | 16.0% |
| Total | 7,709 | – | 11,991 | – | 14,828 | – |
