# Supplementary material for: Greater travel distance to specialized facilities is associated with higher survival for patients with soft-tissue sarcoma: US nationwide patterns
Source: PLoS One. 2021 Jun 4;16(6):e0252381. doi: 10.1371/journal.pone.0252381 (PMC8177553; doi:10.1371/journal.pone.0252381)
Supplement: S5 Table — (DOCX) [file pone.0252381.s009.docx]

| **S5 Table**. Surgical margins in the short/non-academic and very long/academic groups (P < 0.001; chi-square test) | | | | |
| --- | --- | --- | --- | --- |
|  | Short/non-academic | | Very long/academic | |
|  | n | %* | n | %* |
| Negative margin | 5,711 | 66.1% | 1,086 | 74.4% |
| Positive margin | 2,068 | 23.9% | 282 | 19.3% |
| Unknown margin status | 863 | 10.0% | 92 | 6.3% |
| *Data are shown as percentage per number of patients who underwent surgical treatment. | | | | |
